# Successful rechallenge of BRAF/MEK inhibitor with ruxolitinib in BRAF/MEK inhibitor-induced hemophagocytic lymphohistiocytosis like hyperinflammatory syndrome in *BRAF* V600E-mutated lung adenocarcinoma: a case report

**DOI:** 10.3389/fonc.2026.1795399

**Published:** 2026-04-27

**Authors:** Simran Chandra, Sagar Hansraj, Aakriti Adhikari, Ben Ponvilawan, Addison Tolentino, Dhruv Bansal

**Affiliations:** 1 Department of Internal Medicine, University of Missouri–Kansas City School of Medicine, Kansas City, MO, United States; 2 Saint Luke’s Cancer Institute, Kansas City, MO, United States; 3 Division of Hematology and Oncology, Endeavor Health, Evanston, IL, United States

**Keywords:** case report, dabrafenib, hemophagocytic lymphohistiocytosis, ruxolitinib, trametinib

## Abstract

Dabrafenib and trametinib are highly efficacious therapies in patients with *BRAF* V600E-mutated lung adenocarcinoma (LUAD), but they are occasionally associated with the development of hemophagocytic lymphohistiocytosis (HLH). Here, we describe the first reported case of metastatic *BRAF* V600E-mutated lung adenocarcinoma that responded to dabrafenib and trametinib, and subsequently developed treatment-associated HLH-like hyperinflammatory syndrome. With the clinical symptoms and inflammatory markers not being adequately controlled with corticosteroids, the patient ultimately required ruxolitinib for the management of HLH-like hyperinflammatory syndrome while still being maintained on dabrafenib and trametinib for LUAD, achieving sustained disease control for both HLH-like hyperinflammatory syndrome and LUAD. This case illustrates HLH as an uncommon but serious immune-mediated toxicity of BRAF/MEK inhibitors, and co-administration of ruxolitinib as a potentially safe and effective therapy in managing HLH-like hyperinflammatory syndrome triggered by BRAF/MEK inhibitors.

## Introduction

*BRAF* V600E mutation is an established oncogenic driver in a subset of lung adenocarcinoma (LUAD) and is targetable with the combination of BRAF and MEK inhibitors (1). While these agents are generally well-tolerated, rare immune-mediated toxicities such as hemophagocytic lymphohistiocytosis (HLH) may occur (3). HLH is a hyperinflammatory syndrome marked by excessive immune activation and cytokine release, leading to clinical features such as fever, cytopenias, hepatic dysfunction, elevated ferritin, and multiorgan failure (2, 3). HLH has most often been described in association with hematologic malignancies, infections, or immune checkpoint inhibitors, but cases have also emerged with targeted therapies (3–5).

Here, we present a case of HLH-like hyperinflammatory syndrome developing shortly after initiating dabrafenib and trametinib, BRAF and MEK inhibitors, in a patient with LUAD, followed by successful rechallenge of dabrafenib and trametinib in combination with ruxolitinib and low-dose corticosteroids.

## Case presentation

An 89-year-old man with known hypertension, chronic obstructive pulmonary disease, obstructive sleep apnea, hyperlipidemia, atrial fibrillation, and coronary artery disease, was evaluated in the oncology clinic for metastatic lung adenocarcinoma. He was a former smoker with a 30 pack-year history, having quit 40 years earlier.

At initial diagnosis, he had stage IB (*T2aN0M0*) lung adenocarcinoma and underwent a right lower lobe wedge resection. Genomics revealed a *BRAF* V600E Mutation and a PD-L1 (Programmed Death Ligand 1) Tumor proportion score (TPS) 20%. Three years after initial diagnosis, a recurrence in the right hilar thoracic lymph nodes was treated with concurrent chemoradiation, weekly carboplatin, and paclitaxel for six weeks. Five years after initial diagnosis, the cancer recurred in the surgical stump in the right lower lobe of the lung, subaortic lymph nodes, and the right 7^th^-8^th^ ribs.

Given the patient’s advanced age and PD-L1 expression of 20%, single-agent immunotherapy was selected as the treatment approach, with targeted therapy reserved for subsequent lines. He received single-agent pembrolizumab 200 mg every three weeks for 18 months until the cancer progressed after an initial response, with imaging revealing increased size of intrathoracic lymph nodes and new lesions in the left lung apex, the right posterior sulcus of the lung, and the gastrohepatic lymph node.

Dabrafenib and trametinib were initiated approximately six weeks after the last dose of pembrolizumab. He experienced rapid clinical deterioration within two weeks of treatment initiation, with the Eastern Cooperative Oncology Group (ECOG) performance status declining from 1 to 3, and profound fatigue, lack of appetite, and mild fever as the primary symptoms. Laboratory evaluation revealed markedly elevated ferritin levels (4, 501.9 ng/mL), C-reactive protein (CRP) (115 mg/L), and erythrocyte sedimentation rate (ESR) (25 mm/hr), with mild elevations in aspartate aminotransferase (AST) 61 U/L (upper limit of normal 34) and alanine aminotransferase (ALT) 29 U/L (upper limit of normal 49), and normal alkaline phosphatase and bilirubin levels. Thyroid-stimulating hormone level was normal (2.16 µIU/mL). Compared to baseline values prior to starting dabrafenib/trametinib, hematologic and metabolic parameters remained stable or only mildly changed: hemoglobin (11.2 → 12.1 g/dL), white blood cell count (6.28 → 8.9 ×10^9^/L), absolute neutrophil count (4.52 → 7.57 ×10^9^/L), platelets (187 → 174 ×10^9^/L), and creatinine (1.03 → 1.35 mg/dL).

HLH is diagnosed based on a combination of clinical and laboratory criteria, typically requiring fulfillment of at least five of eight criteria as defined in the HLH-2004 guidelines (2).

In this case, although the patient did not meet the full diagnostic criteria (**Table 1**), the constellation of symptoms, marked hyperferritinemia, and lack of an alternative explanation supported a clinical diagnosis of an HLH-like hyperinflammatory syndrome.

**Table 1:** HLH-2004 criteria assessment for the present case.

HLH-2004 Criterion	Yes/No
Fever	Yes
Splenomegaly	No
Cytopenias	No
Hypertriglyceridemia/hypofibrinogenemia	Not assessed
Hemophagocytosis	Not assessed
Ferritin >500	Yes
Elevated soluble CD25	Not assessed
Low NK cell activity	Not assessed

Given the potentially life-threatening nature of HLH, immediate treatment was pursued. Dabrafenib and trametinib were held, and dexamethasone 4 mg twice daily was initiated, leading to rapid symptom improvement and reduction in ferritin to 354.7 ng/mL. The patient was then tapered off steroids over three weeks and rechallenged with a reduced dose of dabrafenib at 75 mg twice daily and trametinib 1 mg daily. Within three weeks after reinitiation, he developed increasing fatigue, along with an elevated ferritin level of 451.9 ng/mL and CRP of 35 mg/L. Prednisone 5 mg daily was initiated, dabrafenib and trametinib were held for two weeks before being resumed at a lower dose of 50 mg twice daily and trametinib 1 mg daily. Prednisone dose was increased to 10 mg daily six weeks later due to rising ferritin levels.

Given the need for steroid-sparing therapy and the goal of maintaining BRAF/MEK inhibition, ruxolitinib, a JAK1/2 inhibitor, was initiated at 5 mg twice daily while continuing prednisone at 10 mg daily. Inflammatory markers improved markedly, with ferritin declining to 261.9 ng/mL from 718.4 ng/mL and CRP to 25 mg/L from 76 mg/L. Dabrafenib and trametinib were continued at reduced doses without further dose modification.

**Figure 1:**
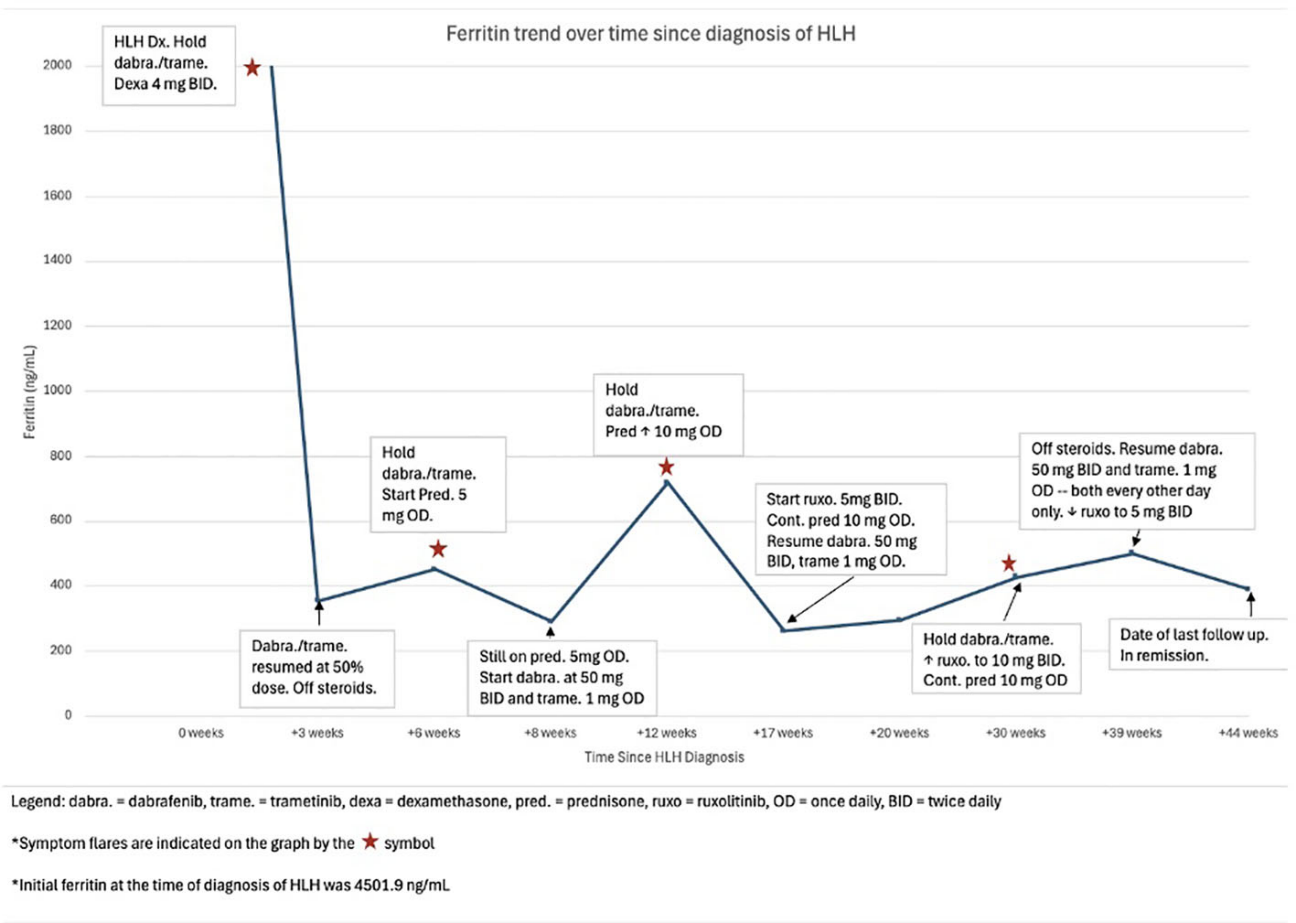
The timeline of clinical course, laboratory values, and medication regimen.

Three months later, a recurrence of fatigue, myalgia, and arthralgia, along with elevated inflammatory markers, prompted another temporary interruption of dabrafenib and trametinib, which was mitigated by increasing the ruxolitinib dose to 10 mg twice daily. Dabrafenib and trametinib were resumed two weeks later at a reduced frequency to every other day, which allowed discontinuation of steroids and reduction of ruxolitinib dose back to 5 mg twice daily. The patient quickly improved and subsequently resumed all therapies without further hyperinflammatory flares. The full timeline of laboratory values and medication dosages is exhibited in **Figure 1** and **Table 2**.

**Table 2:** Clinical course with specific medication dosages and changes over time.

Time since HLH diagnosis	Clinical Events & Treatment	Ferritin (ng/mL)	Dabrafenib	Trametinib	Steroid (prednisone-dose equivalent)	Ruxolitinib
-2 weeks	Started dabrafenib/trametinib for treatment of LUAD		150 mg twice daily	2 mg daily	–	–
0 weeks	HLH diagnosed; dexamethasone started; dabrafenib/trametinib discontinued	4501.9	Held	Held	26.7 mg twice daily	–
+3 weeks	Ferritin improved; off steroids; dabrafenib/trametinib restarted at 50% dose	354.7	75 mg twice daily	1 mg daily	Off	–
+6 weeks	Increased fatigue; CRP and ferritin up; dabrafenib/trametinib held; started prednisone	451.9	Held	Held	5 mg daily	–
+8 weeks	Resumed dabrafenib at lower dose, same dose for trametinib; still on prednisone	290.7	50 mg twice daily	1 mg daily	5 mg daily	–
+12 weeks	Symptoms recurring, ferritin rising; prednisone increased; dabrafenib/trametinib held	718.4	Held	Held	10 mg daily	–
+17 weeks	Ruxolitinib added to allow reinitiation of dabrafenib/trametinib	261.9	50 mg twice daily	1 mg daily	10 mg daily	5 mg twice daily
+20 weeks	Stable on ruxolitinib/steroids, dabrafenib/trametinib	294.2	50 mg twice daily	1 mg daily	10 mg daily	5 mg twice daily
+30 weeks	Symptoms recurred; dabrafenib/trametinib held; ruxolitinib increased	428.2	Held	Held	10 mg daily	10 mg twice daily
+32 weeks	Symptoms improved; resumed dabrafenib/trametinib	418.6	50 mg twice daily every other day	1 mg every other day	10 mg daily	10 mg twice daily
+39 weeks	Off steroids; maintained on dabrafenib/trametinib; ruxolitinib dose reduced	501.8	50 mg twice daily every other day	1 mg every other day	Off	5 mg twice daily
+44 weeks	Last follow-up: patient in remission; on dabrafenib/trametinib and ruxolitinib	389.2	50 mg twice daily every other day	1 mg every other day	Off	5 mg twice daily

At the last follow-up, ten months after the initial HLH-like episode, the patient remained clinically stable from HLH while on dabrafenib 50 mg twice daily every other day, trametinib 1 mg every other day, and ruxolitinib 5 mg twice daily with no steroids. PET/CT scan performed nine months after initiation of dabrafenib and trametinib revealed no imaging evidence of malignancy.

## Discussion

Our case demonstrates a clear temporal association between the initiation of dabrafenib and trametinib and the development of HLH -like hyperinflammatory syndrome, with reproducible clinical flares upon drug rechallenge. HLH remains a rare but increasingly recognized immune-mediated toxicity in patients receiving targeted therapies (3). While the precise incidence is unknown, reports of HLH associated with protein kinase inhibitors, including BRAF and MEK inhibitors, are emerging in both melanoma and lung cancer populations (3). Given its fulminant course and diagnostic complexity, HLH may be underrecognized in the oncology setting and the initial management with corticosteroids alone may be inadequate to support sustained oncologic treatment (4).

**Figure 2:**
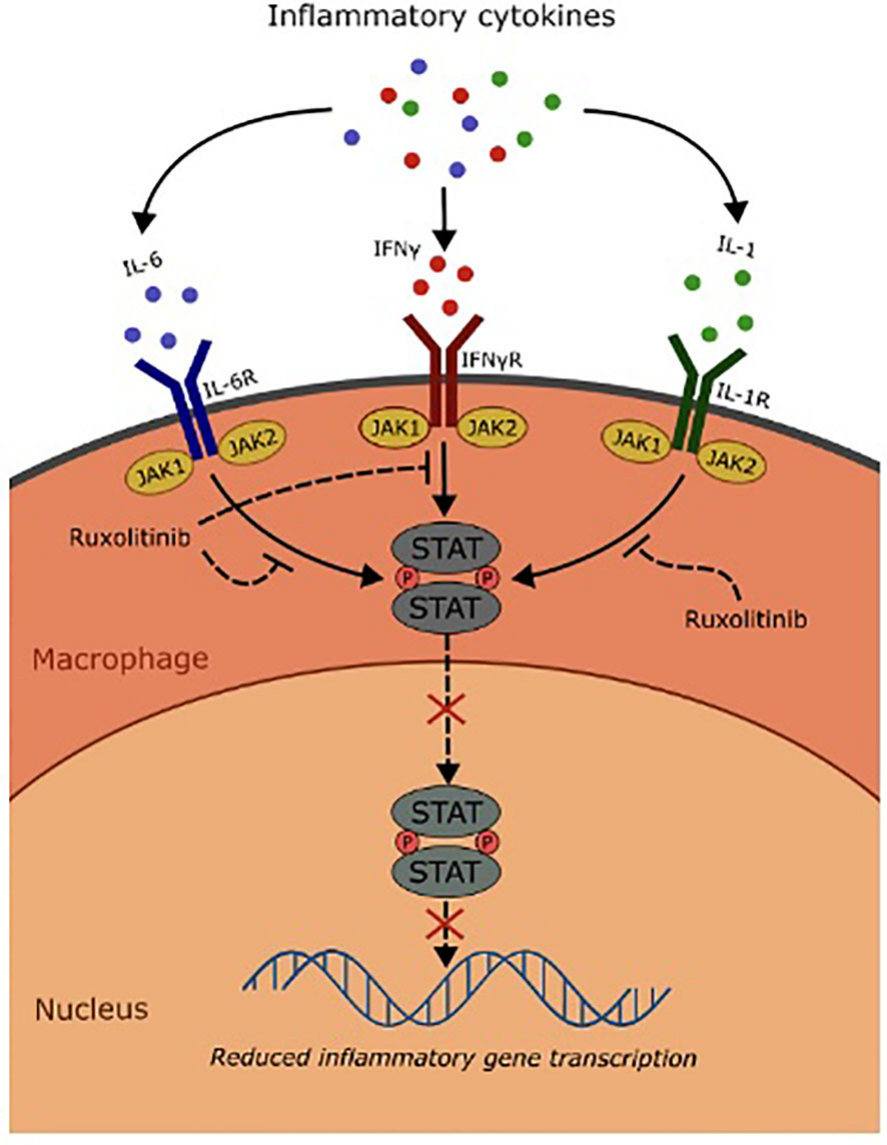
Mechanism of ruxolitinib in the treatment of HLH.

Interestingly, as demonstrated in this case, the introduction of ruxolitinib, a selective JAK1/2 inhibitor, as a steroid sparing agent was pivotal in allowing the continuation of targeted therapies, presumptively via the suppression of inflammatory gene transcriptions downstream from receptor kinases involved in the JAK-STAT pathway, including IFN-γ, IL-6, and IL-1β, which were known to be associated with the pathogenesis of HLH (**Figure 2**) (6). Clinical trials and case series have supported its use in both primary and secondary HLH, with promise as a steroid-sparing agent in patients with malignancy-associated disease (6).

In our patient, ruxolitinib not only improved inflammatory markers but also allowed the continuation of BRAF/MEK inhibitors for primary cancer control without recurrence of HLH-like syndrome, despite multiple prior flares. However, substantial dose reductions of dabrafenib and trametinib were still required to prevent these flares, underscoring that ruxolitinib does not fully eliminate the need to modify the primary therapy.

Ruxolitinib was used as an off-label, exploratory therapy in this case. Current evidence supporting its use in secondary HLH is limited and largely derived from small retrospective cohorts and case series, which suggest potential benefit but remain insufficient to establish definitive efficacy (6). Accordingly, the favorable outcome observed in this patient cannot be attributed solely to ruxolitinib, as dose reduction of the targeted therapy likely also played a critical role.

The patient had previously received pembrolizumab, raising the possibility of delayed immune-related toxicity. Immune checkpoint inhibitors have been associated with HLH, including delayed presentations after treatment discontinuation (7). In addition, prior immune checkpoint blockade may lead to persistent immune activation or remodeling that could potentiate subsequent inflammatory responses (8). However, in this case, the onset of symptoms occurred shortly after initiation of dabrafenib and trametinib, with reproducible flares upon rechallenge, supporting a stronger temporal and causal association with BRAF/MEK inhibition.

Whether HLH in this context represents a drug-specific effect or a broader class effect of BRAF/MEK inhibitors remains unclear, and further studies are needed to better characterize this phenomenon. Because of concerns that HLH might be a class effect, a switch to a different approved combination, such as encorafenib and binimetinib, was not considered for this patient.

The approach of combining dose-reduced targeted therapy with immunosuppression was individualized and guided by clinical response rather than established pharmacokinetic or evidence-based frameworks. While dose modification of BRAF/MEK inhibitors is standard practice for toxicity management, there is limited evidence supporting a structured “low-dose maintenance plus immunosuppression” strategy. As such, this approach should be considered exploratory and hypothesis-generating.

This case adds to the growing literature supporting the use of ruxolitinib in drug-induced HLH. To our knowledge, this is the first report describing the successful use of ruxolitinib to enable long-term continuation of dabrafenib and trametinib in LUAD. Importantly, our experience suggests that with careful dose modulation and immune suppression, BRAF/MEK inhibitor rechallenge may be feasible and safe, even after experiencing HLH-like hyperinflammatory syndrome.

## Conclusion

HLH is a rare but serious complication of BRAF and MEK inhibitors in patients with *BRAF* V600E-mutated LUAD. Ruxolitinib may serve as an effective adjunct to corticosteroids and can facilitate a safe resumption of targeted therapy. Further studies are needed to determine its broader efficacy in drug-induced HLH -like hyperinflammatory syndrome. As such, prompt recognition and initiation of immunosuppression are critical to achieve long-term control of both HLH -like hyperinflammatory syndrome and primary cancer, offering a potential strategy for similar cases in the future.

## Data Availability

The original contributions presented in the study are included in the article/supplementary material. Further inquiries can be directed to the corresponding authors.
